# Molecular Characterization, Tissue Distribution Profile, and Nutritional Regulation of *acsl* Gene Family in Golden Pompano (*Trachinotus ovatus*)

**DOI:** 10.3390/ijms23126437

**Published:** 2022-06-09

**Authors:** Zhigang Yang, Hangbo Zhu, Xiaoping Huang, Aimin Wang, Dizhi Xie

**Affiliations:** 1 Key Laboratory of Freshwater Aquatic Genetic Resources, Ministry of Agriculture, Shanghai Ocean University, Shanghai 201306, China; zgyang@shou.edu.cn (Z.Y.); m200100293@st.shou.edu.cn (H.Z.); 2 Guangdong Laboratory for Lingnan Modern Agriculture, College of Marine Sciences, South China Agricultural University, Guangzhou 510642, China; huangxiaoping@stu.scau.edu.cn; 3 College of Marine and Bioengineering, Yancheng Institute of Technology, Yancheng 224000, China; blueseawam@ycit.cn

**Keywords:** *acsl* gene family, golden pompano, tissue expression, nutritional regulation, LC-PUFA

## Abstract

Long chain acyl-coA synthase (*acsl*) family genes activate the conversion of long chain fatty acids into acyl-coA to regulate fatty acid metabolism. However, the evolutionary characteristics, tissue expression and nutritional regulation of the *acsl* gene family are poorly understood in fish. The present study investigated the molecular characterization, tissue expression and nutritional regulation of the *acsl* gene family in golden pompano (*Trachinotus ovatus*). The results showed that the coding regions of *acsl1*, *acsl3*, *acsl4*, *acsl5* and *acsl6* cDNA were 2091 bp, 2142 bp, 2136 bp, 1977 bp and 2007 bp, encoding 697, 714, 712, 659 and 669 amino acids, respectively. Five *acsl* isoforms divided into two branches, namely, *acsl1*, *acsl5* and *acsl6*, as well as *acsl3* and *acsl4*. The tissue expression distribution of *acsl* genes showed that *acsl1* and *acsl3* are widely expressed in the detected tissues, while *acsl4*, *acsl5* and *acsl6* are mainly expressed in the brain. Compared to the fish fed with lard oil diets, the fish fed with soybean oil exhibited high muscular C_18_ PUFA contents and *acsl1* and *acsl3* mRNA levels, as well as low muscular SFA contents and *acsl4* mRNA levels. High muscular n-3 LC-PUFA contents, and *acsl3*, *acsl4* and *acsl6* mRNA levels were observed in the fish fed with fish oil diets compared with those of fish fed with lard oil or soybean oil diets. High n-3 LC-PUFA levels and DHA contents, as well as the *acsl3*, *acsl4* and *acsl6* mRNA levels were exhibited in the muscle of fish fed diets with high dietary n-3 LC-PUFA levels. Additionally, the muscular *acsl3*, *acsl4* and *acsl6* mRNA expression levels, n-3 LC-PUFA and DHA levels were significantly up-regulated by the increase of dietary DHA proportions. Collectively, the positive relationship among dietary fatty acids, muscular fatty acids and *acsl* mRNA, indicated that *T. ovatus* Acsl1 and Acsl3 are beneficial for the C_18_ PUFA enrichment, and Acsl3, Acsl4 and Acsl6 are for n-3 LC-PUFA and DHA enrichment. The acquisition of fish Acsl potential function in the present study will play the foundation for ameliorating the fatty acids nutrition in farmed fish products.

## 1. Introduction

Long chain fatty acids (LCFAs) are important components of lipids, such as phospholipid and triglyceride, which are the main structural components of membrane and important energy sources for organism. Due to the inertness of LCFAs, they must first be activated to form fatty acyl-CoA by the long chain acyl-CoA synthetase (ACSL) before participating in cellular metabolism [1,2,3]. In mammals, five ACSL isoforms, including ACSL1, ACSL3, ACSL4, ACSL5 and ACSL6, were isolated and identified [4,5]. Additionally, the mammal ACSL isoforms have diverse tissue expression patterns, varying subcellular localization and distinct substrate preferences [2,6].

For example, ACSL1 locates in the mitochondrial membrane and endoplasmic reticulum, and activates preferentially saturated and monounsaturated FAs from C_16_ to C_18_ [2,4]. ACSL3 was found to be localized in the endoplasmic reticulum and lipid droplets, and effectively activates saturated fatty acids [7,8,9]. Several studies revealed that ACSL4 mainly locates in mitochondrial and peroxisome associated membrane, and preferentially activates arachidonic acid (ARA) and eicosapentaenoic acid (EPA) [10,11,12]. ACSL5 localized in mitochondria, which has a substrate preference to linoleic acid [13]. ACSL6 preferentially activates DHA, and participates in the lipogenesis [3,14].

To date, limited study on the physiological function of *acsl* family has been conducted in fish. For example, the study of grass carp (*Ctenopharyngodon idellus*) showed that Acsl1 is involved in activating fatty acids into the β-oxidation pathway and provides energy for fish [15]. In our previous study, the molecular and expression characterization of the *acsl6* cDNA was identified in common carp (*Cyprinus carpio*) and golden pompano (*Trachinotus ovatus*), and explored that Acsl6 performs a critical role in the muscular DHA enrichment of *C. carpio* and *T. ovatus* [16,17]. To compare the physiological functions of the *acsl* gene family in teleost, the molecular and expression characterization of *acsl* family genes were further investigated in golden pompano. These results will provide useful information on the important physiological significance of the *acsl* gene family in teleost.

## 2. Results

### 2.1. Molecular Characterization and Phylogenetic Analyses of acsl Genes

**Figure 1:**
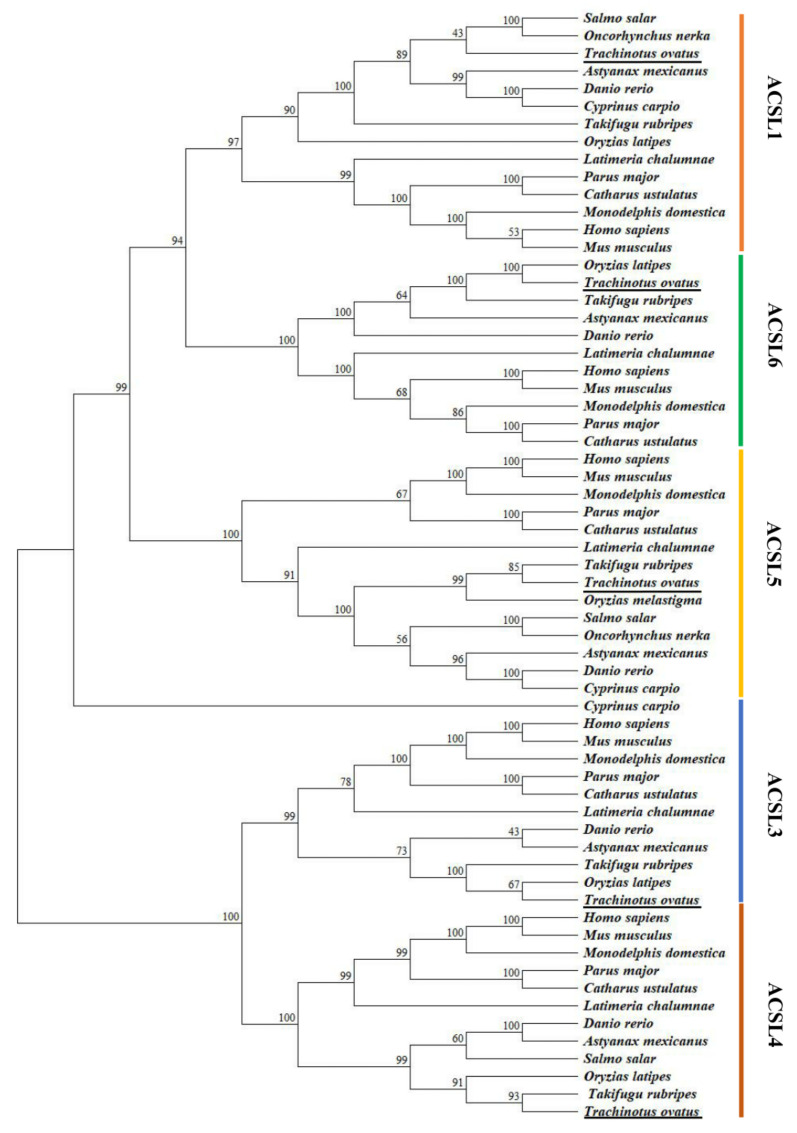
Phylogenetic analysis of the *Trachinotus ovatus* *acsl* family from different animal species. The phylogenetic tree was generated based on an alignment corresponding to the amino acid sequences using ClustalW and MEGA (11.0). The number at nodes represent percentage bootstrap values (only values above 40% are shown) on 1000 replicates.

The full-lengths of *acsl1*, *acsl3*, *acsl4*, *acsl5* and *acsl6* ORF sequences were 2091 bp, 2142 bp, 2136 bp, 1977 bp and 2007 bp, encoding 697, 714, 712, 659 and 669 amino acids, respectively (Figures S1–S5). The phylogenetic tree of Acsl showed that five Acsl clades comprising Acsl1, Acsl3, Acsl4, Acsl5 and Acsl6 are identified, and Acsl3 and Acsl4 form a distinct group from Acsl1, Acsl5 and Acsl6, as well as the fish Acsl sequences being clustered into one branch, while the Acsl sequences of mammal and bird were closely clustered (Figure 1).

### 2.2. Tissue Distribution Pattern of acsl Genes

**Figure 2:**
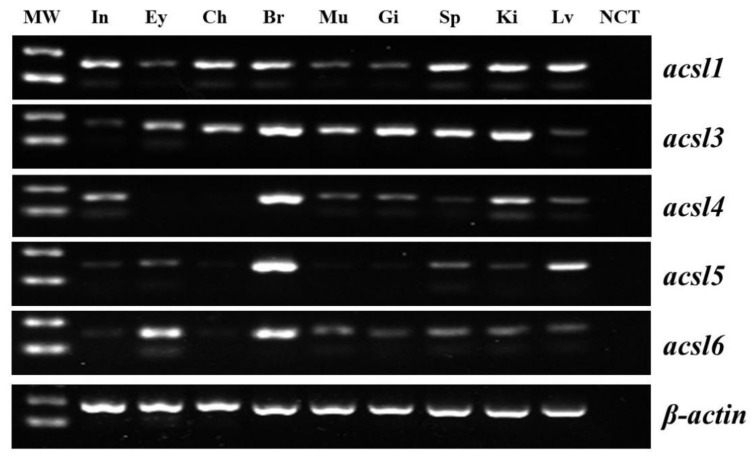
Tissue specificity of *Trachinotus ovatus acsl* family mRNA expression. Expression of the housekeeping gene β-actin is also shown. Abbreviations: MW, molecular weight marker; In, intestine; Ey, eyes; Ch, cholecyst; Br, brain; Mu, muscle; Gi, gill; Sp, spleen; Ki, kidney; Lv, liver; NCT, negative template control.

Among the examined tissues, *T. ovatus acsl1* and *acsl3* genes showed a widespread expression with low *acsl1* transcripts in the eyes, and low *acsl3* transcripts in the intestine and liver (Figure 2). High expression of *acsl4* was detected in the brain and intestine, and with non-detectable level in the eyes and cholecyst. In addition, *T. ovatus acsl5* showed a high expression level in the brain and liver, and with non-detectable level in the cholecyst, muscle and gill. Obviously, the *T. ovatus acsl6* gene was observed in the brain and eyes, followed by the muscle, spleen, kidney, liver and intestine, but not in the cholecyst (Figure 2).

### 2.3. Muscular Fatty Acid Composition and acsl mRNA Level in the Fish Fed Diets with Different Lipid Sources

**Figure 3:**
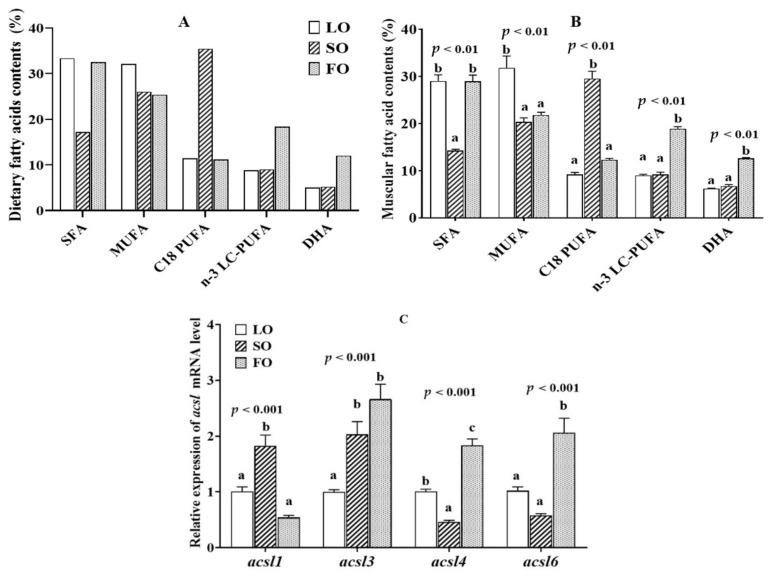
Muscular fatty acid contents (**B**), and *acsl* family mRNA relative level (**C**) of juvenile *Trachinotus ovatus* fed diets with different lipid sources (**A**). Values are means ± SEM from three treatments of fish (*n* = 3) with three fish per cage, and bars not sharing a common letter indicated significant differences (*p* < 0.05) among deletions determined by one-way ANOVA followed by Tukey’s multiple comparison.

The effects of dietary lipid sources on muscle fatty acid composition and *acsl* mRNA expression are shown in Figure 3A–C. The detailed effects of dietary lipid sources on the muscle fatty acid composition were reported in our previous study [17]. The content of saturated fatty acid (SFA) was significantly higher in the fish fed LO and FO diets than that of fish fed SO diets (*p* < 0.05). The fish fed LO diets showed significantly higher muscular monounsaturated fatty acid (MUFA) levels than the other two groups (*p* < 0.05), while higher C_18_ polyunsaturated fatty acids (C_18_ PUFA), and n-3 LC-PUFA and DHA contents were detected in the fish fed with SO and FO diets, respectively (*p* < 0.05). Regarding the changes of muscular *acsl* mRNA expression level (Figure 3C), the expression of *acsl1* mRNA levels in the fish fed SO diets was higher than that in the fish fed LO and FO diets (*p* < 0.05), while a high *acsl3*, *acsl4* and *acsl6* mRNA level was measured in the fish fed FO diets than that of fish fed LO or SO diets (*p* < 0.05).

### 2.4. Muscular Fatty Acid Composition and acsl mRNA Level in the Fish Fed Diets with Different n-3 LC-PUFA Levels

**Figure 4:**
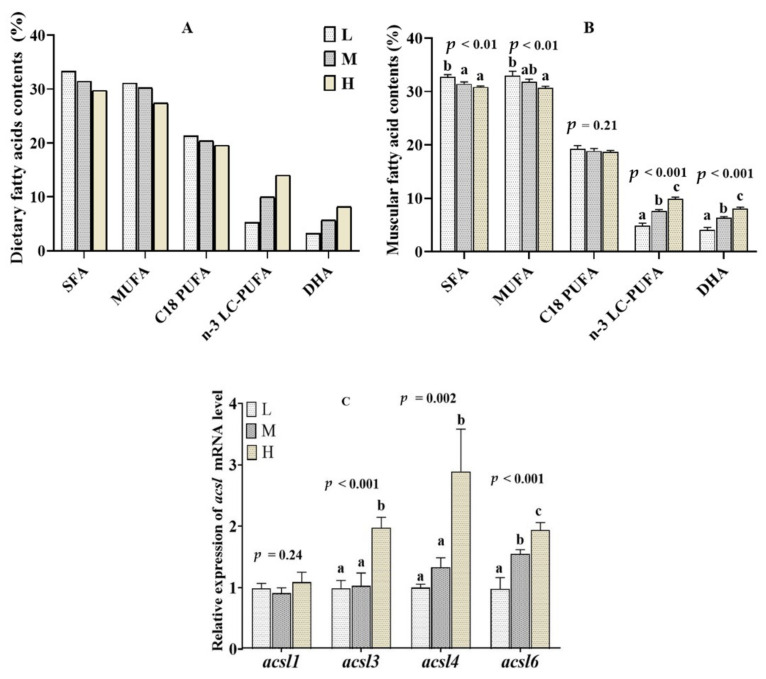
Muscular fatty acid contents (**B**), and *acsl* family mRNA relative level (**C**) of juvenile *Trachinotus ovatus* fed diets with different n-3 LC-PUFA levels (**A**). Values are means ± SEM from three treatments of fish (*n* = 3) with three fish per cage, and bars not sharing a common letter indicated significant differences (*p* < 0.05) among deletions determined by one-way ANOVA followed by Tukey’s multiple comparison.

The fatty acid composition and *acsl* genes expression profile of *T. ovatus* fed diets with different n-3 LC-PUFA levels are shown in Figure 4A–C. The detailed effects of dietary n-3 LC-PUFA levels on the muscle fatty acid composition was reported in our previous study [18]. Briefly, the muscular n-3 LC-PUFA and DHA contents were gradually increased with increasing levels of dietary n-3 LC-PUFA (*p* < 0.05), while the contents of SFA and MUFA decreased accordingly in the muscle (*p* < 0.05) (Figure 4B). Accordingly, the high mRNA expression levels of *acsl3*, *acsl4* and *acsl6* were measured in the muscle of fish fed with high n-3 LC-PUFA diets (*p* < 0.05) (Figure 4C).

### 2.5. Muscular Fatty Acid Composition and acsl mRNA Level in the Fish Fed Diets with Different DHA/EPA Ratios

**Figure 5:**
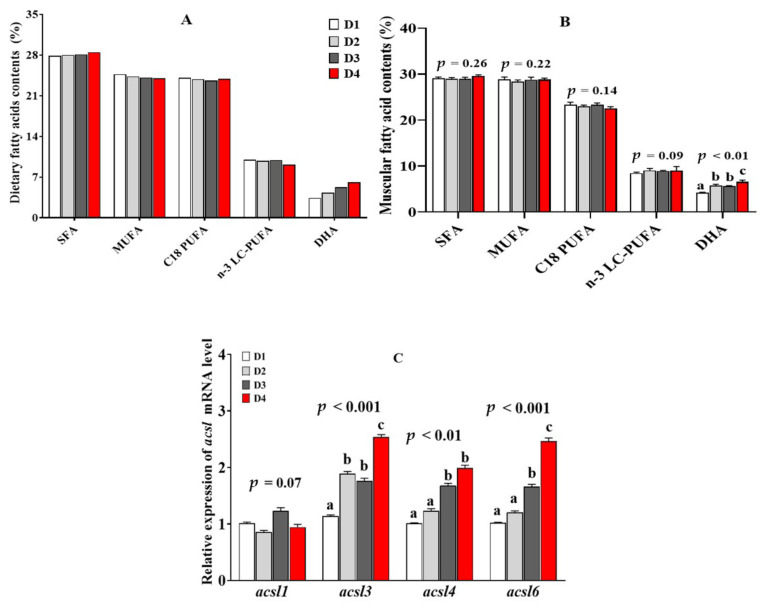
Muscular fatty acid contents (**B**), and *acsl* family mRNA relative level (**C**) of juvenile *Trachinotus ovatus* fed diets with different DHA levels (**A**). Values are means ± SEM from three treatments of fish (*n* = 3) with three fish per cage, and bars not sharing a common letter indicated significant differences (*p* < 0.05) among deletions determined by one-way ANOVA followed by Tukey’s multiple comparison.

The fatty acid composition and *acsl* genes expression profile of *T. ovatus* fed diets with different DHA/EPA ratios are shown in Figure 5A–C. The detailed effects of dietary DHA/EPA ratios on the muscle fatty acid composition were presented in our previous study [19]. In short, the muscle SFA, MUFA, C_18_ PUFA and n-3 LC-PUFA shared comparable levels among the different groups, while the DHA levels showed a gradual upward trend with the dietary DHA/EPA ratios rising (*p* < 0.05) (Figure 5B). Accordingly, the relative mRNA expression of *acsl3*, *acsl4* and *acsl6* in muscle was significantly up-regulated by the increase of dietary DHA proportions (*p* < 0.05) (Figure 5C).

## 3. Discussion

Mammal studies showed that the ACSL1-6 are essential enzymes for the utilization of cellular LCFAs, which is the first enzyme to activate LCFAs, and determines their metabolic fate, including fatty acid *β*-oxidation, lipogenesis and signal lipids [2,20]. For example, ACSL1 and ACSL5 tend to be involved in fatty acids *β*-oxidation [21,22]. ACSL3 and ACSL4 mediate the lipid synthesis and lipid droplets biogenesis [23,24]. ACSL6 preferentially activates DHA, and mediates the synthesis of lipids such as TAG and phospholipids [14]. In the present study, the molecular characterization, tissue distribution and fatty acids regulation of *acsl* genes were investigated from the marine teleost, *T. ovatus*, which provides evidences for their potential roles in the muscle fatty acid metabolism.

The phylogenetic evolutionary analysis of the Acsl family shows that five Acsl clades comprising Acsl1, Acsl3, Acsl4, Acsl5 and Acsl6 are identified, and Acsl3 and Acsl4 form a distinct group from Acsl1, Acsl5 and Acsl6, which is consistent with previous reports of vertebrates [2,25]. In addition, the different Acsl isoforms showed diverse tissue distribution patterns. For example, the *T. ovatus acsl1* gene was widely expressed in intestine, brain, muscle and liver, which is consistent with reports in mammals [26] and grass carp [15]. The *acsl3* has widespread expression in the intestine, brain, muscle, spleen and kidney of *T. ovatus*, zebrafish (*Danio rerio*) and mammals [2,27]. While *T. ovatus* and zebrafish *acsl4*, and mammal *Acsl4* have a high expression in the brain and intestine, and with non-detectable level in the eyes and cholecyst [2,3]. *T. ovatus* and zebrafish *acsl5* is mainly distributed in the brain, spleen, kidney and liver, which has similar results to zebrafish. In contrast, mammal *Acsl5* is mainly expressed in the intestine, with less expression in the liver and kidney [28,29]. Additionally, mammalian *Acsl6* is strictly expressed in the brain and testis [2,14], while a widely widespread distribution pattern of the *acsl6* gene was found in the analyzed tissues of *T. ovatus*, zebrafish and grass carp [2,16].

Similar to the diversity of distribution in tissues, ACSL isoforms are also diverse in their preference for fatty acid substrates [4,5]. Studies in mammals have found that ACSL1 preferentially activates saturated and monounsaturated fatty acids, from C_16_ to C_18_ [2]. Over-expression of ACSL1 promoted the binding of oleic acid to diacylglycerol and phospholipids in rat liver cells and the internalization of oleic acid in PC12 neuronal cells [30]. Depletion/over-expression of ACSL3 caused a significant reduction/increase in the oleic acid absorption of mammal cells [27,31], ACSL3 exhibits a preference for C_18_-C_20_ PUFAs over saturated and monounsaturated fatty acids [32]. *T. ovatus acsl1* and *acsl3* mRNA expression was increased significantly in SO diets (enriched with C_18_ PUFA), suggesting that Acsl1 and Acsl3 are closely related to C_18_ PUFA metabolism. Furthermore, dietary n-3 LC-PUFA levels and DHA/EPA ratio had no significant effect on the *acsl1* mRNA expression, and a lower expression of *acsl1* transcripts was found in fish fed FO diets than that of fish fed LO and SO diets. The results suggested that *T. ovatus* Acsl1 hardly plays any role in activated n-3 LC-PUFA. Similarly, the *acsl1* mRNA expression was significantly down-regulated in grass carp fed FO diets [15]. The study of Atlantic salmon (*Salmo salar*) showed a down-regulation of *acsl* (the specific gene isoforms is not given) expression in the fish fed diets with high EPA + DHA levels [33]. Consistent with the induced *acsl1* and *acsl3* mRNA abundance, the muscle C_18_ PUFA levels in the *T. ovatus* fed SO diets were significantly increased compared with the LO and FO diets. The results speculated that *T. ovatus* Acsl1 and Acsl3 may be involved in the metabolism of muscular C_18_ PUFA.

Interestingly, an up-regulation of *acsl3* mRNA expression and n-3 LC-PUFA level was observed in the muscle of *T. ovatus* fed with FO diets (enriched with n-3 LC-PUFA), high dietary n-3 LC-PUFA levels or high DHA ratio, suggestive of increased activity of n-3 LC-PUFA metabolism. Similarly, the hepatic *acsl3* mRNA expression was increased in grass carp fed high n-6 LC-PUFA diets [34]. In mammals, ACSL3 was found to preferentially activate EPA and mediates lipid droplet formation [32], which provides acyl-CoA for glyceride synthesis on the surface of lipid droplets [35]. Therefore, the results suggested that *T. ovatus* Acsl3 may also be involved in the metabolism and deposition of intracellular n-3 LC-PUFA.

In addition to *acsl3*, both *T. ovatus acsl4* and *acsl6* mRNA were also increased in the FO, high n-3 LC-PUFA level and high DHA ratio groups, which is consistent with the up-regulation of very long chain acyl-CoA synthetase (*acsvl*) transcript levels in the Atlantic salmon and grass carp fed dietary docosahexaenoic acid and fish oil diets, respectively [36,37]. Numerous mammal studies have shown that both ACSL4 and ACSL6 have a preference for the activation of n-3 LC-PUFA [14]. ACSL4 is closely associated with the incorporation of EPA and DHA into membrane phospholipids [38], and could improve the intramuscular EPA and DHA content [39]. The enhanced expression of mammal *Acsl6* also promoted the deposition of DHA in the skeletal muscle [40], brain [14], spermatids and seminiferous tubules [41,42]. It is noteworthy that the expression of *T. ovatus acsl3*, *acsl4* and *acsl6* mRNA was significantly positively correlated with dietary DHA ratio, but not with dietary EPA proportions. However, in mammals, ACSL3 [32] and ACSL4 [39,42] are also involved in activating the cellular EPA. We speculate that the difference may be due to the abundance of DHA in fish. The obvious differences observed in their mRNA expression that responded to dietary DHA and EPA proportions might suggest that *T. ovatus* Acsl3, Acsl4 and Acsl6 perform greater contribution to the enrichment of DHA than EPA.

In conclusion, five isoforms of the *acsl* genes family were identified in *T. ovatus*, which is divided into two branches, namely, *acsl1*, *acsl5* and *acsl6*, and *acsl3* and *acsl4*. *T. ovatus acsl* gene isoforms that showed diverse tissue distribution, such as *acsl1* and *acsl3*, are widely expressed in the detected tissues, while *acsl4*, *acsl5* and *acsl6* are mainly expressed in the brain. The positive relationship among dietary fatty acids, muscular fatty acids and *acsl* mRNA suggested that *T. ovatus* Acsl1 and Acsl3 are beneficial for the C_18_ PUFA enrichment, and Acsl3, Acsl4 and Acsl6 for n-3 LC-PUFA enrichment (especially for DHA enrichment). The results provided useful information for exploring the physiological significance of fish *acsl* genes.

## 4. Materials and Methods

### 4.1. Ethical Statement

All experimental operations were performed according to the procedures of the Ethics Committee of Animal Experiments of the South China Agricultural University (SCAU) and approved by the Ethics Committee of Animal Experiments of SCAU (SCAU-AEC-2010-0416).

### 4.2. Animal Experiments

To compare the substrate preference and potential function of Acsl isoforms in *T. ovatus*, the nutritional regulation of fatty acids on the expression of *acsl1*, *acsl3*, *acsl4*, *acsl5* and *acsl6* was investigated, and three culture experiments were performed. In culture experiment Ⅰ, using fish meal, fermented soybean meal and soybean meal as protein sources, and lard oil (LO, rich in SFA), soybean oil (SO, rich in C_18_ PUFA) or fish oil (FO, rich in LC-PUFA) as lipid sources, three isoproteic (about 45.00% crude protein) and isolipidic (about 12.00% crude lipid) diets were formulated [17]. In culture experiment Ⅱ, taking fish meal, fermented soybean meal and soybean protein concentrate as protein sources, and soybean oil, rapeseed oil, perilla oil and DHA- and EPA-enriched oil as lipid sources, three isoproteic (about 44.50% crude protein) and isolipidic (about 12.30% crude fat) diets with different n-3 LC-PUFA levels (5.25%, 10.05% and 14.05%, namely, L, M and H) were prepared [18]. In culture experiment Ⅲ, using fish meal, fermented soybean meal and soybean meal as protein sources, and palm oil, soybean oil and DHA- and EPA-enriched oil as lipid sources, four isoproteic (about 46.81% crude protein) and isolipidic (about 12.50% crude lipid) diets with different DHA/EPA ratios (0.53, 0.81, 1.17 and 2.12, namely, D1, D2, D3 and D4) were formulated [19]. The detailed dietary formulations and proximate and fatty acid compositions were shown in our previous studies [17,18,19].

Each experimental diet was fed to *T. ovatus* juveniles in triplicate cages for 8 weeks. After the end of the feeding experiments, muscle from three fish per cage (nine fish per dietary group) was sampled to analyze the fatty acid composition and *acsl* genes expression.

### 4.3. Trachinotus Ovatus acsl Genes Cloning

Total RNA was extracted from various tissues of golden pompano (*Trachinotus ovatus*) by using TRizol reagent (Invitrogen, Carlsbad, CA, USA). The purified total golden pompano RNA was reverse-transcribed into cDNA by using the AMV reverse-transcriptase first-strand cDNA synthesis kit. In order to amplify the open reading frame (ORF) of the *acsl* cDNAs, gene-specific primers complementary to the *acsl1*, *acsl3*, *acsl4*, *acsl5* and *acsl6* genes were designed according to the whole genome sequencing data of golden pompano (Table 1). PCR was performed using an RT-PCR kit from TAKARA (Takara, Maebashi, Japan). The amplification procedure consisted of 35 cycles of denaturation at 94 °C for 30 s, annealing at 52 °C for 30 s and extension of 2 min at 72 °C. PCR positive fragments were confirmed by DNA sequencing (Shanghai Sangon, Shanghai, China).

### 4.4. Phylogenetic Analysis

The neighbor-joining (NJ) method was used to analyze the sequence phylogeny of Acsls based on the amino acid sequences from seven fish species (*Salmo salar*, *Oncorhynchus nerka*, *Astyanax mexicanus*, *Cyprinus carpio*, *Takifugu rubripes*, *Oryzias latipes*, *Latimeria chalumnae*), two bird species (*Parus major*, *Catharus ustulatus*) and three mammal species (*Monodelphis domestica*, *Mua musculus*, *Homo sapiens*). The confidence of the phylogenetic tree-branch topology of Acsls was performed through 1000 bootstrap iterations.

### 4.5. Tissue Distribution Pattern of Trachinotus ovatus acsl Genes

Tissue samples, including liver, eye, intestine, cholecyst, brain, gills, muscle, spleen and kidney, were collected from three juvenile fishes (sex could not be distinguished by means of naked eye) to investigate the tissue distribution pattern of *acsl* genes. All tissues were immediately frozen in liquid nitrogen and kept at −80 °C until used.

Total RNA was extracted from these above tissue samples, and 1 μg RNA of each sample was reverse transcribed into cDNA using the first-strand cDNA synthesis kit (Invitrogen, Carlsbad, CA, USA). PCR protocol: 94 °C for 4 min, 94 °C for 30 s, 55 °C for 30 s, 72 °C for 45 s, 30 cycles and finally 72 °C for 5 min.

### 4.6. Quantitative Real-Time PCR Analysis

Using the genes-specific primers described in Table 1, the effects of dietary DHA level on the expression of the *acsl* genes family were analyzed by real-time PCR using the Light Cycler 480 system (Roche, Basel, Switzerland). The qPCR operations were performed according to the protocol previously described [17]. β-actin was selected as the reference gene. Each sample had at least three replicates. The relative expression levels were determined by 2^−ΔΔCT^ method.

### 4.7. Fatty Acid Compositions Evaluation

The detection of dietary and muscle fatty acid compositions were performed according to the method described previously [17]. Briefly, total dietary and muscle lipids were extracted using a mixture of chloroform/methanol (*v*/*v*, 2:1) containing 0.01% butylated hydroxytoluene [43]. The fatty acid methyl esters (FAME) of total lipid were performed using boron trifluoride diethyl etherate (ca. 48%, Acros Organics, Waltham, MA, USA). The FAME were analyzed using gas chromatography (GC-2010 plus; Shimadzu, Kyoto, Japan), and individual FAME were identified through comparison with commercial standards (Sigma, St. Louis, MO, USA), and quantified by CLASS-GC2010-plus workstation (Shimadzu).

### 4.8. Statistical Analysis

Except for the dietary proximate and fatty acid compositions, all data were presented as mean ± SE (standard error, *n* = 3), and analyzed by one-way analysis of variance (ANOVA), followed by Tukey’s multiple comparison. A significant difference level was considered at *p* < 0.05. All analyses were performed using SPSS version 19.0 (SPSS Inc., Chicago, IL, USA).

## Figures and Tables

**Table 1:** PCR primers sequence used in this study.

Subject	Primer Name	Primer Sequence (5′-3′)	References/ Accession Number
RT-PCR	RT-*acsl1*-F	ATGCAGGCTCAGGAAGTCCTGAGAC	PRJNA574781
	RT-*acsl1*-R	TTAGATCTTAATTTTAGAATAAAGT	
	RT-*acsl3*-F	ATGAAGCTGAAGGAGGACCTGAA	PRJNA574781
	RT-*acsl3*-R	TTATTTTCCACCGTACATTCTCTC	
	RT-*acsl4*-F	ATGGGTCTCCAGGCAGACTCAAC	PRJNA574781
	RT-*acsl4*-R	TTATTTGCCCCCATACATCCTCT	
	RT-*acsl5*-F	ATGGAATTCCTTTTCCAGTTGCTC	PRJNA574781
	RT-*acsl5*-R	TTATTGGATGTTAGCATATAGTT	
	RT-*acsl6*-F	ATGCTCGCATTCGTTTTGGTCTC	MN481524
	RT-*acsl6*-R	TCACATGGAGATGCTGCTGTAGA	
qPCR	*acsl1*-F	CTGAAGATCGTGGACAGGAAGAAGC	PRJNA574781
	*acsl1*-R	CAACCACACAGGAAGTCAGGGTCAG	
	*acsl3*-F	TGCCTATGCCAACAGTGACCAGTC	PRJNA574781
	*acsl3*-R	ATCGCTCCAGTTTCGCTGAGATAGC	
	*acsl4*-F	AGGCAAGGACACGCTGGATAAG	PRJNA574781
	*acsl4*-R	TCCAGTTCATTGTAGGACAGCCA	
	*acsl6*-F	GCCTCGTTGAGCGCGGCAAGGGCT	MN481524
	*acsl6*-R	AAGCCTGAGAAATCAGCTACCACG	
	*β-actin*-F	TACGAGCTGCCTGACGGACA	Chen et al., 2022
	*β-actin*-R	GGCTGTGATCTCCTTCTGC	

## Data Availability

Data supporting reported results can be asked to the authors.

## Supplementary Materials

Supplementary materials can be downloaded at: https://www.mdpi.com/article/10.3390/ijms23126437/s1.

## Abbreviations

ACSL	Long chain acyl-coA synthase
ARA	Arachidonic acid
DHA	Docosahexaenoic acid
EPA	Eicosapentaenoic acid
LCFAs	Long chain Fatty acids
LC-PUFA	Long chain-polyunsaturated fatty acid
MUFA	Monounsaturated fatty acid
SFA	Saturated fatty acid
