# Supplementary material for: Molecular Characterization, Tissue Distribution Profile, and Nutritional Regulation of acsl Gene Family in Golden Pompano (Trachinotus ovatus)
Source: Int J Mol Sci. 2022 Jun 9;23(12):6437. doi: 10.3390/ijms23126437 (PMC9224283; doi:10.3390/ijms23126437)
Supplement: Supplementary file 1 [file ijms-23-06437-s001.zip › ijms-1739465-supplementary.pdf]

ATGCAGGCTCAGGAAGTCCTGAGACAGCTGCGCATTCCCGAGCTGGATGACGTTCGGCAGTACGTGCGCGGCTTG

M Q A Q E V L R Q L R I P E L D D V R Q Y V R G L

CCCACCAACGCGCTCATGGGTATGGGTGCTTTCGCTGCCATCACAACCTACTGGTTTGCAACCCGGCCAAAAGCC

P T N A L M G M G A F A A I T T Y W F A T R P K A

CTCAAACCGCCCTGTGACCTCAGTCTGCAGTCGGTGGAAATACCAGGTGGAGAACGTGCACGAAGATCAATGCTG

L K P P C D L S L Q S V E I P G G E R A R R S M L

AACGACAGTGAGATAAACATGACGCACTACTACAGCGATGCATGCACAATGTACGAGGTGTTCAGGCGAGGACTC

N D S E I N M T H Y Y S D A C T M Y E V F R R G L

AGAGTATCGAATAATGGACCTTGTCTCGGATCGAGAAAACCAAACCAGCCATACGAGTGGCAGTCTTACAGAGAG

R V S N N G P C L G S R K P N Q P Y E W Q S Y R E

GTGGCAGACAGAGCAGAGTACATCGGCTCTGCTCTCCTTCACCGAGGACACTCTCACACAGGAGACAAGTTCGTT

V A D R A E Y I G S A L L H R G H S H T G D K F V

GGTATTTTTTCCCAGAATAGGCCAGAGTGGACCATTTCAGAGCTGGCCTGTTACACATACTCCTTGGTGGCAGTC

G I F S Q N R P E W T I S E L A C Y T Y S L V A V

CCACTGTACGACACACTCGGCACAGAGGCCATTGACTACATTATCGACAAAGCTGCCATCTCAACCGTGATCTGC

P L Y D T L G T E A I D Y I I D K A A I S T V I C

GATGTCCCTGAAAAGGCTGGGATGATTCTGGACTGCATCAGCGGGAAAACCCGAACTGTCAAGACGATCGTGCTC

D V P E K A G M I L D C I S G K T R T V K T I V L

ATGGAGGCGTTTGACAGCGACCTGGTGACCCGTGGACAGGAGTGCGGCATTGAGATCCTGAGCTTGAAGGACTTT

M E A F D S D L V T R G Q E C G I E I L S L K D F

GAGGCCTTGGGTAAAGCAAACCACCAGACACCAGTGCCCCCTAAGCCAGAGGACCTCGCACTCATCTGCTTTACG

E A L G K A N H Q T P V P P K P E D L A L I C F T

TCTGGAACCACAGGAAACCCGAAAGGTGCAATGCTTACTCATGGAAATGTAATCTCCAACACTGCAGCTTTCATT

S G T T G N P K G A M L T H G N V I S N T A A F I

AAAGTAACAGAGGTAAACTGCATGCTGAACCTCCATGACATTCATGTATCCTATCTCCCCCTAGCTCACATGTTT

K V T E V N C M L N L H D I H V S Y L P L A H M F

GAGAGGGTTGTACAGGGGGTCATCCTCATCCACGGGGCCCGAATCGGCTACTTCCAAGGGGACATTCGACTTTTA

E R V V Q G V I L I H G A R I G Y F Q G D I R L L

ATGGACGATTTGAAGACGCTGCAACCAACAGTCTTCCCTGTGGTCCCACGTCTCCTTAACCGCATGTTTGATAAG

M D D L K T L Q P T V F P V V P R L L N R M F D K

GTATTTGGTCAGGCCAATACACCGCTGAAGAGATGGGTGCTGGACTTCGCCTTCAGGAGGAAGGAGGCAGAGCTG

V F G Q A N T P L K R W V L D F A F R R K E A E L

AAAAATGGCGTGGTCAGAAAGGACAGCATGTGGGACAAACTCATCTTCAGAAAAGTACAGGCGAGTCTGGGCGGT

K N G V V R K D S M W D K L I F R K V Q A S L G G

CGTGTGAGACTGATGATTACAGGAGCAGCACCGGTGTCACCGACCATCCTGACGTTCCTGCGAGCTGCTCTGGGC

R V R L M I T G A A P V S P T I L T F L R A A L G

TGTCAGTTTTATGAAGGCTATGGTCAAACTGAATGTACAGCTGGGTGCTCCATGTCAATGCCTGGAGACTGGACA

C Q F Y E G Y G Q T E C T A G C S M S M P G D W T

GCAGGTCACGTCGGGCCTCCTCTGCCCTGCAACATTATCAAACTGGTGGATGTGGCAGAAATGAATTACCTGGCA

A G H V G P P L P C N I I K L V D V A E M N Y L A

GCCAATGGAGAGGGAGAGGTGTGTGTCAAAGGACCAAATGTATTCCAGGGATACCTGAAAGACCCCGAGAAAACA

A N G E G E V C V K G P N V F Q G Y L K D P E K T

GCCGAGGCAATCGACAAGGATGGATGGCTGCACACAGGCGACATTGGGAAATGGCTTCCTAATGGCAGTCTGAAG

A E A I D K D G W L H T G D I G K W L P N G S L K

ATCATTGACAGAAAGAAGCACATTTTCAAGCTGGCACAGGGTGAATACATCGCCCCCGAGAAAATAGAGACCATC

I I D R K K H I F K L A Q G E Y I A P E K I E T I

TATAATCGCAGTGATCCAGTGGCCCAGATATTTGTACATGGTGATAGCTTACAGGCATGCCTGGTGGGATAGTG

Y N R S D P V A Q I F V H G D S L Q A C L V G I V

GTGCCTGATCCAGACTTTTTACCTATTTGGGCAAAGAAAAAGGGAATTGAAGGAACCTACTCTGAATTATGCAAT

V P D P D F L P I W A K K K G I E G T Y S E L C N

AGCAAGGAGGTGAAGAACGCCATTCTGGAGGACATCCTGCGGCTGGGCAAAGAAGCAGGGCTAAAGTCGTTTGAA

S K E V K N A I L E D I L R L G K E A G L K S F E

CAGGTGAGAGATATTGCATTACGCACCGAGATGTTTACCGTCCAGAACGGTCTCCTGACACCCACCCTGAAGGCC

Q V R D I A L R T E M F T V Q N G L L T P T L K A

AAGAGGGCTGAGCTTCGGAGCCGCTTCAGAGAGCAGATAGATGAACTTTATTCTAAAATTAAGATCTAA

K R A E L R S R F R E Q I D E L Y S K I K I

Figure S1. Complete nucleotide and putative amino acid sequences of open reading frame of *Trachinotus ovatus acsl1*.

ATGAAGCTGAAGGAGGACCTGAACCCTGTGGTGCTGTTCCTCTTCCACCTCGTGGTGTGGATCTACACCCTCATC

M K L K E D L N P V V L F L F H L V V W I Y T L I

ACTTTCCTGCCTTACCACCTCTTCAGCTCGGTGTCGATGCCCGTCGGGGGCGTCTTCGGCTCAGAGGAGGAGCGA

T F L P Y H L F S S V S M P V G G V F G S E E E R

GCCAAGAGAGCCAAGGCCCGATCTGTGCTGGGATGTCCCGAGGGGCCCTACAGAGCGGTGAGTGCCACCAAGAGG

A K R A K A R S V L G C P E G P Y R A V S A T K R

CTGGTGACGTCGACGCACCCGGGGGTGGACACCCTGGACAAAATGTTCGAATACGCAGCAACGAGGTTCCCGCAA

L V T S T H P G V D T L D K M F E Y A A T R F P Q

AGAGACTGTCTCGGCACGAGGGAGGTGATCAGTGAGGAGGACGAGCAGCAGAGCAATGGGAAGGTGTTTAAGAAG

R D C L G T R E V I S E E D E Q Q S N G K V F K K

GTAGTGCTGGGTGAGTACCGCTGGCGCTCCTATGAGGAGGTCCTCACAGCGGCATCCCAGCTGGGCAGCGGTCTG

V V L G E Y R W R S Y E E V L T A A S Q L G S G L

GCGTCACTGGGTCAGCAGCCGAAGCACAACATCGCCATCTTCTGTGAGACGCGAGCCGAGTGGATCATAGCTGCC

A S L G Q Q P K H N I A I F C E T R A E W I I A A

CAGGCCTGCTTCATGTACAACTTCCCAACTCTTTACTCCACCCTGGGAGGTCCAGCCATCGCTCACGGGCTGAAT

Q A C F M Y N F P T L Y S T L G G P A I A H G L N

GAGACTCAGGTCACCCACATCATCACCAGCAGAGAGCTTCTGGAGACCAGACTCAAGATCCTAATAGAAGTTCCA

E T Q V T H I I T S R E L L E T R L K I L I E V P

AGGCTGCAGCATATCATTGTAGTGGATAACACACCAACTTCATGGCCCGGGTATCCACGTGGCATCAGCGTCCAC

R L Q H I I V V D N T P T S W P G Y P R G I S V H

AACATGGCTGCTGTTCAGAAACTGGGGGCCAGGTCTGAGAATGCACGTGAGCGCAAGCAGCCGCTGCCTTCAGAC

N M A A V Q K L G A R S E N A R E R K Q P L P S D

ATTGCAGTCATCATGTACACCAGCGGATCCACGGGCATACCAAAGGGAGTCATGATCTCCCATAGCAACATCATC

I A V I M Y T S G S T G I P K G V M I S H S N I I

GCCGGCATCACAGGGATGGCTGAGCGGATACCTAACCTGAGTGAGGAGGACACCTACATCGGCTACCTGCCTCTC

A G I T G M A E R I P N L S E E D T Y I G Y L P L

GCCCACGTACTGGAGCTCAGTGCAGAGCTCGTATGTATTTCCCATGGCTGTAGAATCGGCTACTCCTCACCTCAA

A H V L E L S A E L V C I S H G C R I G Y S S P Q

ACCCTCGCTGACCAGTCAACCAAGATCAAGAAAGGAAGTAAGGGAGACACCAGCATCCTGCAGCCCACCCTGATG

T L A D Q S T K I K K G S K G D T S I L Q P T L M

GCGGCCGTTCCGGAGATCATGGATCGCATCTATAAGAACGTGATGACCAAAGTTGAGGAGATGAGTTACGTCCAG

A A V P E I M D R I Y K N V M T K V E E M S Y V Q

CGTACTCTCTTCATTCTGGCTTACAACTACAAGCTGGAGCAGTTCACCAAAGGATACAGCACACCTCTATGTGAC

R T L F I L A Y N Y K L E Q F T K G Y S T P L C D

AGGCTGGTGTTCAGGAAAGTTCGATCTCTGCTGGGAGGTCGGACGCGAGTCTTGTTGTCTGGAGGAGCGCCGCTC

R L V F R K V R S L L G G R T R V L L S G G A P L

TCCGCGGCCACACAGCGTTTCATGAATGTGTGTTTCTGCTGCCCAGTGGGTCAGGGATACGGCCTGACGGAGACT

S A A T Q R F M N V C F C C P V G Q G Y G L T E T

TGTGGAGCTGGAACCATCAGCGAATGGGATTACAGCACTGGGAGAGTGGGAGGGCCGCTGGTCTGCTGTGAGATA

C G A G T I S E W D Y S T G R V G G P L V C C E I

AAGCTCAAAGACTGGGTGGAGGGTGGTTATCGGAGCACAGACAAGCCACATCCCAGAGGAGAGATTCTGATTGGT

K L K D W V E G G Y R S T D K P H P R G E I L I G

GGACCGAATGTAACCATGGGATACTACAAGAACGAGGCCAAAAACCAAGGAGACTTCTTCGTGGGTGAGAACGGC

G P N V T M G Y Y K N E A K N Q G D F F V G E N G

CAGCGGTGGTTCTGCACAGGAGACATCGGAGAGTTTCACGAAGATGGCTGCCTCAAGATAATCGATCGTAAAAAA

Q R W F C T G D I G E F H E D G C L K I I D R K K

GACTTGGTGAAGCTGCAGGCGGGAGAGTACGTCTCCCTAGGAAAGGTGGAGGCCATGTTGAAGAATTGCTCGCTG

D L V K L Q A G E Y V S L G K V E A M L K N C S L

GTCGACAACATCTGTGCCTATGCCAACAGTGATGAGACATATGTGATTGGCTTTGTGGTGCCCAATCAGAAGCAG

V D N I C A Y A N S D E T Y V I G F V V P N Q K Q

CTGCAGGCTCTGGCCGACCAGTACAGCATCCGAGGCTCATGGGAGGAGCTGTGCAACAGCAAGGCCATTGAGGAG

L Q A L A D Q Y S I R G S W E E L C N S K A I E E

CTGGTCCTCAAGGTCATCACTGAAGCTGCACTCGCAGCCCAGCTAGAGCGCTTTGAGATTCCTCGCAAAATCCGC

L V L K V I T E A A L A A Q L E R F E I P R K I R

CTGAGCCCAGACCCCTGGACCCCTGAGACAGGATTAGTGACTGATGCATTCAAGCTCAAGCGCAAGGAGCTGAAA

L S P D P W T P E T G L V T D A F K L K R K E L K

ACACATTACCAGGATGACATTGAGAGAATGTACGGTGGAAAATAA

T H Y Q D D I E R M Y G G K

Figure S2. Complete nucleotide and putative amino acid sequences of open reading frame of *Trachinotus ovatus acsl3.*

ATGGGTCTCCAGGCAGACTCAACGCTCCAATCTATCCTCCTCTTTCCAATCCACCTTCTGATATGGCTGTACTCC

M G L Q A D S T L Q S I L L F P I H L L I W L Y S

GTCCTGTCCTTCCTACCCTGGTACTACATCACCGGTGCTGGGGAAAGACAAGCTTTGTCCAAGCGGATAAAGGCC

V L S F L P W Y Y I T G A G E R Q A L S K R I K A

CGTTCCACTTCAGGCTGTGCTGAGGGACCATACCGCTCTGTGGACCACTTCGACTGCCTGGCCAGGGAGGACTTC

R S T S G C A E G P Y R S V D H F D C L A R E D F

CCAGGCAAGGACACGCTGGATAAGCTTTTTGAACATGCTGTACAGCGATTTGGACAAGCACACTGTCTCGGCACC

P G K D T L D K L F E H A V Q R F G Q A H C L G T

CGAGATATTCTGAGTGAGGAGAATGAGGTTCAGCCCAGTGGTAAAGTATTTAAAAAGCTGATCCTGGGGGAGTAT

R D I L S E E N E V Q P S G K V F K K L I L G E Y

AAATGGCTGTCCTACAATGAACTGGATTCTATAGTCAGCGAGTTTGGCAGTGGATTGGCAGCTCTCGGACAACAG

K W L S Y N E L D S I V S E F G S G L A A L G Q Q

CCCAAAAGCACTATTGCAATCTTCTGTGAAACCAGAGCGGAGTGGATGATCACTGCCCAGGCGTGCTTCAGGCGC

P K S T I A I F C E T R A E W M I T A Q A C F R R

AATTTCCCATTGGTGACATTCTATGCCACACTCGGAGAGGAGGCTATTGCTTTTGGACTGAATGAGACCGGTGTT

N F P L V T F Y A T L G E E A I A F G L N E T G V

ACTCATCTAGTCACCAGTGTGGAACTGCTTGAGACTAGGCTGAAAAATGTGCTTCCACAGATCCCAAAACTGAAG

T H L V T S V E L L E T R L K N V L P Q I P K L K

CATGTGATCTATGTAGACCAGAAGAAAGTGCGCACAGAAGGCTACCCAGCAGGACTCTCCATCCACAGCATGCAG

H V I Y V D Q K K V R T E G Y P A G L S I H S M Q

GCTGTACGAGAGCTGGGCGCGCTGCCTGAAAATATCGGGAGGGCAATCGTGAAGCCCCAGCCTGCCGATCTGGCT

A V R E L G A L P E N I G R A I V K P Q P A D L A

GTGGTGATGTACACCAGTGGCTCCACAGGCAGACCCAAAGGAGTCATGATTGTCCACAGTAACCTGATCGCAGGA

V V M Y T S G S T G R P K G V M I V H S N L I A G

ATGACAGGACAGTGTGAGCGCATCCCTGGACTCGGGCCTGATGATACCTACATAGCCTATCTGCCTCTGGCTCAT

M T G Q C E R I P G L G P D D T Y I A Y L P L A H

GTTCTGGAAATGACAGCTGAAATCTCCTGTGTCACATATGGCTGTCGGATCGGCTACTCATCCCCGCAGACACTG

V L E M T A E I S C V T Y G C R I G Y S S P Q T L

TCAGACCAGTCCACCAAGATTAAGAAAGGAAGTAAAGGAGACTGCTCAGTGCTCAGACCCACCCTGATGGCAGCT

S D Q S T K I K K G S K G D C S V L R P T L M A A

GTGCCAGAAATTATGGATCGCATCAACAAAAATGTGATGAGCAAAGTGCAGGAAATGAGCTTCATTCAGAAGACT

V P E I M D R I N K N V M S K V Q E M S F I Q K T

CTGTTTACACTGGGCTACAAGTATAAACTGGAGCAGATCAAGCGGGGCTATGACGCACCACTCTGCAATGCCCTG

L F T L G Y K Y K L E Q I K R G Y D A P L C N A L

TTATTCCGGAAAGTCAAGAAGCTGCTGGGCGGAAGAGTGAGGATGATGTTGTCGGGAGGGGCCCCCCTGTCTTCA

L F R K V K K L L G G R V R M M L S G G A P L S S

GCCACTCAGAGATTTATGAATGTATGTTTCTGTTGTCCAGTGGGCCAGGGCTATGGCCTCACTGAAACCTGTGGA

A T Q R F M N V C F C C P V G Q G Y G L T E T C G

GCAGGCACCATCACAGAGGTTGCAGACATCAGCACCGGCCGTGTTGGAGCTCCTCTTATTTGCTGTGAGGTCATA

A G T I T E V A D I S T G R V G A P L I C C E V I

CTCAGAGACTGGGCTGAAGGTGGCTACACCAGGAAGGACAAGCCGAACCCAAGAGGAGAGATCCTGATTGGTGGT

L R D W A E G G Y T R K D K P N P R G E I L I G G

CCCAATGTAACCATGGGTTACTACAGGAATGAAAGCAAGGATCAGGACTTTTTCGTGGATGAAAAAGGCCAGAGG

P N V T M G Y Y R N E S K D Q D F F V D E K G Q R

TGGTTCTGCACTGGTGATGTCGGAGAGATTTACCCAGATGGTTGTCTACAAATAGTGGACCGCAAGAAAGACTTG

W F C T G D V G E I Y P D G C L Q I V D R K K D L

GTCAAACTGCAGGCTGGGGAGTATGTGTCTCTTGGTAAAGTCGAGTCTGCGCTGAAAAACTGCTCTCTCATAGAC

V K L Q A G E Y V S L G K V E S A L K N C S L I D

AACATCTGTGCTTACGCAAACAGTGACCAGAACTATGTGATCAGCTTTGTGGTTCCCAACCAGAAGAAGTTGACA

N I C A Y A N S D Q N Y V I S F V V P N Q K K L T

GAACTGGCCAAACAGAGAAGCATCGTTGGAGCATGGGAGGAGATCTGCACTCACCCCGAAATGGAAAGAGAAGTT

E L A K Q R S I V G A W E E I C T H P E M E R E V

CTGAAGGAGATCAAAGTCGTCGCTGCTAACATTAAACTCCAGCGATTTGAGATTCCAGTGAAGGTGCATCTGAGT

L K E I K V V A A N I K L Q R F E I P V K V H L S

CCAGAGCCGTGGACCCCAGAGACGGGTCTTGTGACGGATGCGTTCAAGCTGAAGAGGAAAGAGCTGAAGAATCAC

P E P W T P E T G L V T D A F K L K R K E L K N H

TATCTCCACCACATAGAGAGGATGTATGGGGGCAAATAA

Y L H H I E R M Y G G K

Figure S3. Complete nucleotide and putative amino acid sequences of open reading frame of *Trachinotus ovatus acsl4*.

ATGGAATTCCTTTTCCAGTTGCTCTTCTCCCCGCTCCCGACCCCAGCCATCATCTCCCTGTTCGCCCTGGCAGCC

M E F L F Q L L F S P L P T P A I I S L F A L A A

GCCACCTTGTTTTACCTCAACACACGACCCAGTCCCCTCCGCACTCCGATTGACCTCAAACACCAAACTGTGGGG

A T L F Y L N T R P S P L R T P I D L K H Q T V G

ATCAAGGATGGAGCAAGAAAGACCGCTTTGCTTGAGGGTAACAACAACCTGATGTCATACTGCTATGAAGACGCC

I K D G A R K T A L L E G N N N L M S Y C Y E D A

AAGACCATCTATGAGGTTTTTCAGAGGGGGCTGAAAGTTTCAGGTAATGGTCCATGCCTGGGCTACAGAAAACCA

K T I Y E V F Q R G L K V S G N G P C L G Y R K P

GGAAGGCCGTACCAGTGGCTGAAGTACAAACAGGTGTCTGACAGAGCGGAGCACCTGGGGTCAGGGCTGCTTCAC

G R P Y Q W L K Y K Q V S D R A E H L G S G L L H

AGAGGTCTGAAGCCAAACCAAGACACTTTTATTGGCATCTTTGCCCAGAATAGACCTGAATGGATTATTGGTGAA

R G L K P N Q D T F I G I F A Q N R P E W I I G E

CTGGCCTGTTACACCTACTCCATGGTAGCGGTTCCCCTGTACGACACCCTGGGTCCTGAGGCTCTTGTGTTCATT

L A C Y T Y S M V A V P L Y D T L G P E A L V F I

ATCGCGGAGATCTCCACGGTGCTTTGTGACAATCAGAAGAAGGCAGAAACACTGCTGCAGAACCGGGAGAAAGGC

I A E I S T V L C D N Q K K A E T L L Q N R E K G

CAGAGTCTTGTTCTCAAAACCATCGTCATCATGGACCCTTTCAACTCCGAGTTGGTCGAGAGAGGAACAAAGTGT

Q S L V L K T I V I M D P F N S E L V E R G T K C

GGGGTGGATGTCGTGTCCATGCAGGATGTGGAGGCTCTGGGGAAAAGTAATCTTCAAAAACCAGGAGACCCCAAG

G V D V V S M Q D V E A L G K S N L Q K P G D P K

GGCGCGATGCTGACCCATGAGAATGTGGTCTCTGATGCTGCAGGTGTCATCAAAAGCTTTGAGGTTCCAAGTACC

G A M L T H E N V V S D A A G V I K S F E V P S T

CAGGATGTCAGCATTTCATTCCTGCCTTTAGCTCACATGTTTGAGAGAGTTGTCCAGACGGTGGTATATGGTGCT

Q D V S I S F L P L A H M F E R V V Q T V V Y G A

GGGGCTAGGGTGGGATTCTTCCAGGGCGATATCAGACTGCTGCCAGATGACATGAAAACCCTGCAGCCCACCATT

G A R V G F F Q G D I R L L P D D M K T L Q P T I

TTCCCAGTAGTTCCTCGACTTCTCAACCGTGTCTATGACAAAGTCCAGAGCGGAGCAAAGACTCCGTTCAAGAAA

F P V V P R L L N R V Y D K V Q S G A K T P F K K

TGGCTTCTGAACTTTGCTGTGGAGAGGAAATATGCTGAAGTCAAGGAGGGCATCATCAGGAACAACAGCATATGG

W L L N F A V E R K Y A E V K E G I I R N N S I W

GATAAGCTCATCTTCCACAAAGTCCAGGAGTCTTTGGGGGGACGTGTGCGGATCATGGTGACCGGAGCAGCTCCC

D K L I F H K V Q E S L G G R V R I M V T G A A P

ATATCTCCCTCTGTTCTCAACTTCCTCAGGGCGGCTCTGGGCTGTCAGATCTTTGAGGGGTACGGCCAGACTGAG

I S P S V L N F L R A A L G C Q I F E G Y G Q T E

TGCACAGCTGGCTGCACCTTCACCATGCCAGCAGACGCCACCACAGGGCATGTTGGGGTGCCACTGCCTTGTAAT

C T A G C T F T M P A D A T T G H V G V P L P C N

ATTGTGAAGTTGGTGGATGTTGAAGAAATGAATTACTTTGCTTCAAATGGTGAAGGAGAGGTCTGTATCAAGGGT

I V K L V D V E E M N Y F A S N G E G E V C I K G

AGAAATGTGTTTAAGGGATACTTGAAAGACCCAGAGAAGACTGCAGAGGCCATGGATAAAGATGGTTGGCTCCAC

R N V F K G Y L K D P E K T A E A M D K D G W L H

ACTGGGGATATTGGAAAATGGCTTCCGTCTGGGGTTCTGAAGATCATCGACCGGAAGAAGAATATCTTCAAGCTG

T G D I G K W L P S G V L K I I D R K K N I F K L

GCTCAGGGGGAGTACATCGCTCCAGAGAAGATTGAGAATGTGTATGTGCGCAGTGGACCTGTGGCCCAGGTGTTT

A Q G E Y I A P E K I E N V Y V R S G P V A Q V F

GTGCATGGAGACAGCCTACAGTCTTGCCTGGTTGCCATTGTGGTCCCTGATCCAGAGATACTGCCAGGGTTTGCC

V H G D S L Q S C L V A I V V P D P E I L P G F A

AAAAATCTGGGGTGCCAAGGCTCCATTGAAGAACTCTGCAAAAATGAAATTAAGAAAGCAATCCTTTCAGACATG

K N L G C Q G S I E E L C K N E I K K A I L S D M

ACCAAACTGGGTAAAGAAGCAGGACTCAAATCCTTTGAGCAGGTGAAAGACCTGTACCTCCATCCAGAGCAGTTC

T K L G K E A G L K S F E Q V K D L Y L H P E Q F

ACCATTGAGAACGGTCTGTTAACTCCCACGCTCAAGGCCAAGAGGGCCGAGCTCAAAACTCTCTTCCAGCCACAG

T I E N G L L T P T L K A K R A E L K T L F Q P Q

ATCGACAAACTATATGCTAACATCCAATAA

I D K L Y A N I Q

Figure S4. Complete nucleotide and putative amino acid sequences of open reading frame of *Trachinotus ovatus acsl5.*

ATGGAGAAGATGCAGGCCCAGGAGATGATGAGCGGCCTGAGGATACCGGAGATGGACGATATCGGCCAGTTCTTC

M E K M Q A Q E M M S G L R I P E M D D I G Q F F

CGCTCCCTGCCAACATCCACGCTGGTGGGCATCGGTGCTCTGACAGCCGTGCTGGCGTACTGGTTGGCCACTAGA

R S L P T S T L V G I G A L T A V L A Y W L A T R

CCCCGCCCCATCAAACCACCCTGCAGCCTTCTGCACCAGTCTGAGGAGGTGCCGGAAGATGGAGGTCGCAGGTCC

P R P I K P P C S L L H Q S E E V P E D G G R R S

ATGATGGGCGATAGTTCCAAGCTGCTGAGTCATTACCATGACGACGCCAGGACCATGTACGAGGTCTTCCAGAGA

M M G D S S K L L S H Y H D D A R T M Y E V F Q R

GGCCTCCACATATCTGGTGATGGACCTTGCTTAGGCTCACGGCTCCCTAACCAGCCTTACAAATGGATGTCCTAC

G L H I S G D G P C L G S R L P N Q P Y K W M S Y

AAAGAGGTGACAGCCCGGGCTGAACATCTGGGTTCAGGCTTGTTGCACCAAGGCTGCCAGCCCAACCCCAACCAG

K E V T A R A E H L G S G L L H Q G C Q P N P N Q

TTCATAGGAGTGTTTGCCCAGAACAGGCCAGAGTGGATCATCTCAGAGCTAGCTTGCTACACCTACTCCATGGTG

F I G V F A Q N R P E W I I S E L A C Y T Y S M V

GTGGTTCCTCTTTATGACACGCTGGGCCCAGACGCCATACGGTTCATCATTAATACAGCTGACATCTCCACGGTC

V V P L Y D T L G P D A I R F I I N T A D I S T V

ATCTGCGACAAAGTAGAGAAAGCTGAGGTGCTGCTGGATAACGTGGAGCACAAGGAGACTCCGGCCCTTCGAAGA

I C D K V E K A E V L L D N V E H K E T P A L R R

ATCATCCTGATGGATGCCTTTGACGCTGGCCTCGTTGAGCGCGGCAAGGGCTGCAGCGTCCATGTGCAGGCCATA

I I L M D A F D A G L V E R G K G C S V H V Q A I

CAGGAAGTGGAGGCTCTGGGCAGAGAGCACTACAGAAAACCTATACCACCAGCGCCGGACGACCTGTCTATTGTG

Q E V E A L G R E H Y R K P I P P A P D D L S I V

TGTTTCACCAGTGGAACCACAGGAAACCCAAAGGGAGTCATGCTCACCCATGGGAACGTGGTAGCTGATTTCTCA

C F T S G T T G N P K G V M L T H G N V V A D F S

GGCTTCCTCAAAGTAACAGATAAAGTCATTTTCCCCAACCAAGATGATTGCCTAATTTCCTTCCTGCCACTGGCC

G F L K V T D K V I F P N Q D D C L I S F L P L A

CACATGTTTGAGAGGCTCATCGAGTCTGTGGTGTACTGCCATGGAGGACGAATCGGCTTCTATCAGGGTGACATC

H M F E R L I E S V V Y C H G G R I G F Y Q G D I

CGTCTTCTCCCAGATGACATGAAGGCCCTTCGGCCTACCATTTTCCCTGTGGTGCCTCGTCTGCTCAACCGCATG

R L L P D D M K A L R P T I F P V V P R L L N R M

TATGACAAGATCTTCAGCCAGGCTAACAGCCCGTTAAAACGTTGGCTGCTCAACTTTGCTGCTAAGAGAAAAGGT

Y D K I F S Q A N S P L K R W L L N F A A K R K G

GCTGAAGTCAGTAGCGGCATCATACGCAGCGACAGCATCTGGGACAAAATCTTCTTCAGTAAGATTCAGGTGTAC

A E V S S G I I R S D S I W D K I F F S K I Q V Y

GAGGCGTATGGCCAGACAGAGTGCACAGCTGGCTGCACCTTCACCACACCTGGAGACTGGACCCCATTAGGTCAT

E A Y G Q T E C T A G C T F T T P G D W T P L G H

GTCGGGGCTCCGCTGCCATGTAACCTTATTAAACTGGTGGACGTTCCTGAGAAGAACTACTTTGCCTCGAAGGGA

V G A P L P C N L I K L V D V P E K N Y F A S K G

GAGGGAGAGGTCTGTGTGAAGGGACCGAACGTGTTTAAGGGCTACCTCAAAGACCCAGAGAGGACAGCCGAGACG

E G E V C V K G P N V F K G Y L K D P E R T A E T

CTAGATGCAGACGGCTGGCTCCACACCGGAGACATTGGAAAATGGTTACCTAATGGCACGCTGAAGATCATTGAC

L D A D G W L H T G D I G K W L P N G T L K I I D

AGGAAGAAGCATATCTTCAAGTTGGCGCAGGGCGAGTACATCTCCCCAGAGAAGATCGAGAACATCTACATTAGG

R K K H I F K L A Q G E Y I S P E K I E N I Y I R

AGTGAACCTGTGGCACAGCTCTATGTCCATGGAGATAGTCTGCAGTCCTGTTTGGTGGGCATTGTAGTTCCTGAC

S E P V A Q L Y V H G D S L Q S C L V G I V V P D

CCTGAAGTCATGCCCGAATGGGCCAAGAAGAAAGGCATATTAGGTACCTACAAGGACCTCTGTAAAAACACGGAA

P E V M P E W A K K K G I L G T Y K D L C K N T E

CTTAAGAAAGCAATCCTTGAAGATTTGGTGCGTCTGGGCAAGGCCAGTGGCCTCCACTCCTTTGAGCAGGTTAAG

L K K A I L E D L V R L G K A S G L H S F E Q V K

AACATCTACATCCACAACGAGATGTTCTCCATTGAGAACGGCCTCCTGACTCCAACACTAAAGGCCAAGAGACCG

N I Y I H N E M F S I E N G L L T P T L K A K R P

GAGCTGAAGGAGTTCTTCAAGGGGAAGATCGAACAGCTCTACAGCAGCATCTCCATGTGA

E L K E F F K G K I E Q L Y S S I S M

Figure S5. Complete nucleotide and putative amino acid sequences of open reading frame of *Trachinotus ovatus acsl6.*
